# From Gut to Lungs: The Hidden Respiratory Impacts of IBD: A Systematic Review of the Literature

**DOI:** 10.3390/ijms26188912

**Published:** 2025-09-12

**Authors:** Ionela Preotesoiu, Luana Alexandrescu, Bogdan Cimpineanu, Ioan Tiberiu Tofolean, Ionut Valentin Stanciu, Alexandra Herlo, Eugen Dumitru, Daria Maria Alexandrescu, Elena Dina, Cristina Daniela Aftenie, Andreea Nelson Twakor, Doina Ecaterina Tofolean

**Affiliations:** 1 Doctoral School, Faculty of General Medicine, “Ovidius” University, 900470 Constanta, Romania; ionelia.phb@yahoo.com; 2 Gastroenterology Department, “Sf. Apostol Andrei” Emergency County Hospital, 145 Tomis Blvd., 900591 Constanta, Romania; ioan.tofolean@365.univ-ovidius.ro (I.T.T.); eugen.dumitru@yahoo.com (E.D.); elena.dina@ymail.com (E.D.); afteniecristina@gmail.com (C.D.A.); 3 Faculty of General Medicine, “Ovidius” University, 900470 Constanta, Romania; ionutstvalentin@gmail.com (I.V.S.); tofoleandoina@yahoo.com (D.E.T.); 4 Nephrology Department, “Sf. Apostol Andrei” Emergency County Hospital, 145 Tomis Blvd., 900591 Constanta, Romania; 5 Pneumology Department, “Sf. Apostol Andrei” Emergency County Hospital, 145 Tomis Blvd., 900591 Constanta, Romania; 6 Department XIII, Discipline of Infectious Diseases, “Victor Babes” University of Medicine and Pharmacy Timisoara, 2 Eftimie Murgu Square, 300041 Timisoara, Romania; alexandra.mocanu@umft.ro; 7 Faculty of Medicine, Titu Maiorescu University, 040051 Bucharest, Romania; alexandrescu_daria@yahoo.com; 8 Internal Medicine Department, “Sf. Apostol Andrei” Emergency County Hospital, 145 Tomis Blvd., 900591 Constanta, Romania; andreea.purcaru@365.univ-ovidius.ro

**Keywords:** inflammatory bowel disease, pulmonary involvement, Crohn’s disease, ulcerative colitis, systematic review, extraintestinal manifestations

## Abstract

Pulmonary complications are an important yet underappreciated aspect of inflammatory bowel disease (IBD), which includes Crohn’s disease (CD) and ulcerative colitis (UC). These conditions often manifest with extraintestinal symptoms that can significantly influence the clinical trajectory of the disease. Pulmonary involvement in IBD can range from mild symptoms, such as a persistent cough, to severe conditions, including interstitial lung disease or pulmonary embolism. This systematic review aims to assess the prevalence, clinical presentations, and implications of pulmonary involvement in IBD patients. A comprehensive literature search was conducted using PubMed database up to the 1st of May 2024. Inclusion criteria focused on studies involving adult IBD patients with documented pulmonary symptoms, evaluated through clinical, radiological, and histopathological approaches. Of the 463 studies identified, 27 met the inclusion criteria, consisting of 36,264 patients. Pulmonary manifestations were classified into airway diseases and parenchymal involvement. Airway diseases, including bronchiectasis and chronic bronchitis, were the most common, followed by parenchymal conditions such as organizing pneumonia and interstitial lung disease (ILD). Smoking was identified as a significant risk factor for pulmonary involvement. Pulmonary involvement in IBD is diverse and often underdiagnosed. Early recognition and management are crucial to improving patient outcomes.

## 1. Introduction

### 1.1. Epidemiology

**Figure 1:**
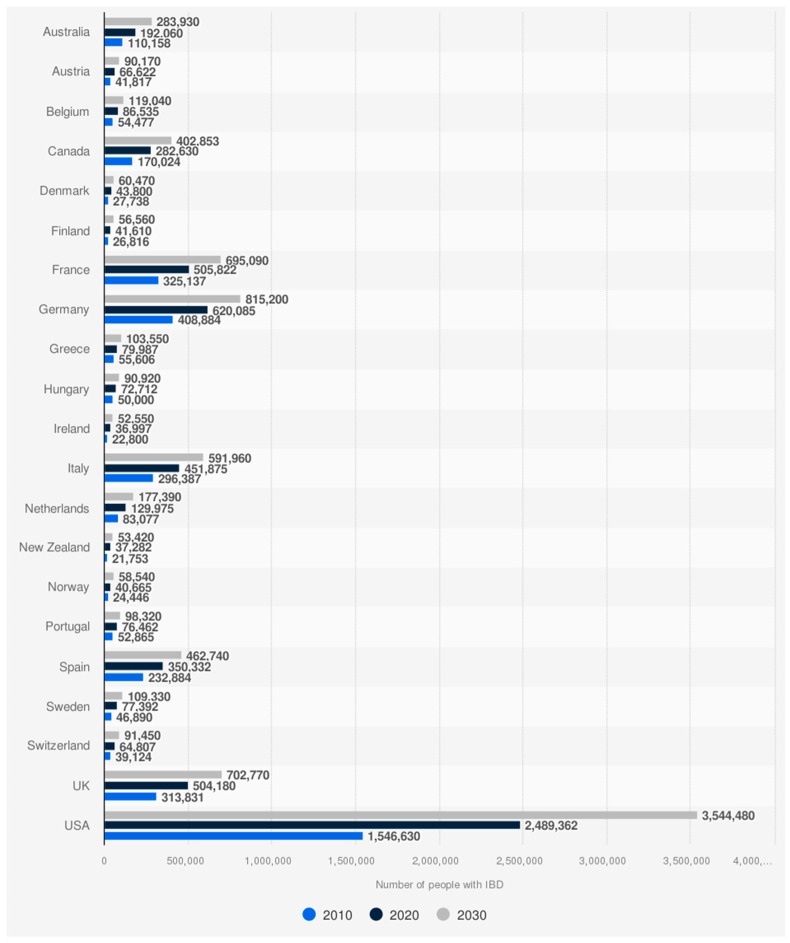
Global rise in IBD cases: 2010–2030 projections [4].

Ulcerative colitis and Crohn’s disease are the primary types of chronic inflammatory bowel disorders that include recurring periods of inflammation and remission [1]. The prevalence of UC and CD has seen a steady rise in the recent years. Global Data’s epidemiology forecast predicts that the number of diagnosed prevalent cases of UC is projected to rise from around two million cases in 2023 to 2.1 million cases by 2031 [2]. Similarly, the diagnosed prevalent cases of CD are expected to increase from approximately 1.6 million cases in 2023 to 1.7 million cases by 2032 in eight major countries, which include the US, 5EU (France, Germany, Italy, and Spain), Japan, and Canada [3]. Figure 1 shows the 2010–2030 rise in IBD projections.

In developed nations, IBDs have become a significant issue in the field of gastroenterology. Additionally, there has been a concerning increase in the occurrence of these diseases in regions that traditionally had low incidence rates, such as East Asia, the Indian subcontinent, the Middle East, Latin America, and Eastern Europe [5].

### 1.2. Pulmonary Involvement

**Figure 2:**
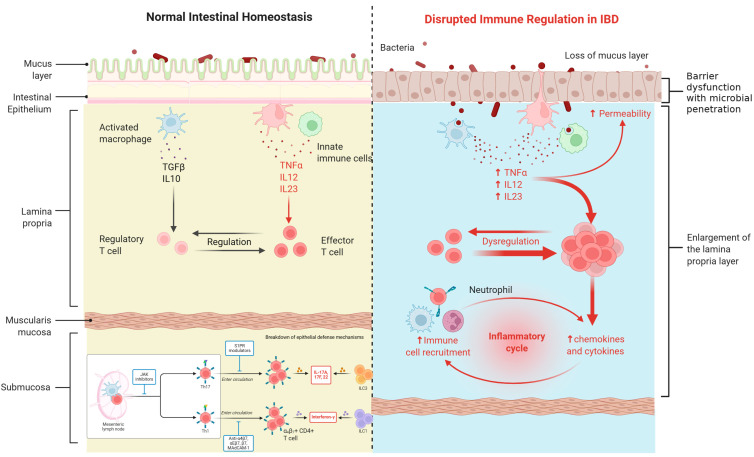
Immunological landscape in normal and IBD-affected gut mucosa. Created with Biorender [8].

While the gastrointestinal tract is primarily impacted, both UC and CD are systemic inflammatory illnesses that often affect organs outside the gastrointestinal tract [6]. Systemic manifestations may appear several years after the onset of bowel disease and can impact nearly all organs, including the musculoskeletal, hepatobiliary, cardiovascular, renal and genitourinary, pancreatic, nervous, and bronchopulmonary systems [2]. The extraintestinal symptoms of inflammatory bowel disease often align with the progression of the disease and significantly affect the patients’ quality of life, morbidity, and even mortality [7]. Figure 2 below shows the disruption of immune regulation in IBD.

According to available data, the prevalence of IBD in 2021 was 321.2 cases per 100,000 people [9]. This is an increase of 46% compared to the prevalence of 200 cases per 100,000 people in 2006 [9].

The first identification of pulmonary involvement in individuals with inflammatory bowel disease was made by Kraft et al. almost four decades ago [9]. Both UC and CD may impact any region of the respiratory system. Patients with IBD might have a range of respiratory abnormalities, such as malfunction in the small and large airways, as well as obstructive and interstitial lung diseases [10]. Respiratory asymptomatic IBD patients may be studied using several methods such as screening studies, high-resolution computed tomography (HRCT), bronchoscopy, histological analysis, and pulmonary function tests (PFTs) [11]. Such methods help to identify the early alterations in the airways of these patients. These modifications, including the subclinical changes in the peripheral airways and inflammation in the lung tissue, may not be identified with regular CT scans and pulmonary function tests [12].

### 1.3. Pathophysiological Mechanisms

**Figure 3:**
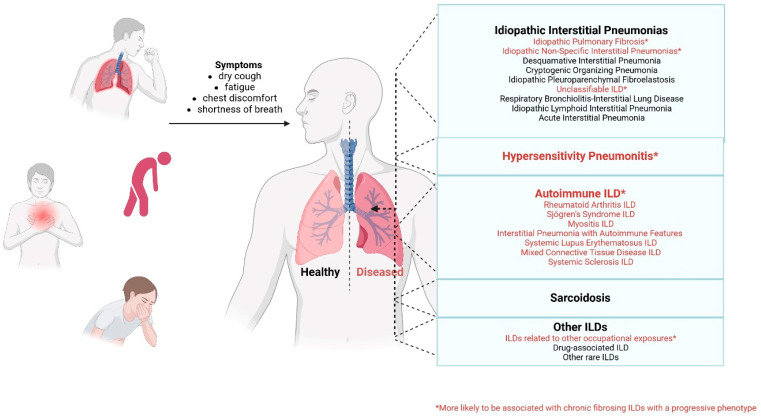
Overview of interstitial lung diseases: classification and symptoms. Created with BioRender [8].

Patients experiencing active disease often demonstrate a more obvious pulmonary dysfunction compared to those in remission [13]. Among the various pulmonary manifestations, ILDs are particularly concerning due to their association with high morbidity and mortality rates [14]. These disorders can present in diverse forms, including nonspecific interstitial pneumonia (NSIP), organizing pneumonia (OP), and even progressive pulmonary fibrosis [15]. Although the exact mechanisms linking IBD and ILD are still under investigation, research suggests that shared inflammatory pathways and immune system dysregulation play a significant role in their co-occurrence (see Figure 3) [16,17].

Airway inflammation not only compromises patients’ quality of life but also significantly contributes to increased morbidity and mortality [18]. For instance, Luo et al. demonstrated that intestinal mucosal inflammation could trigger distal airway inflammation [19]. Similarly, research by Lui et al. on animal models highlighted a direct connection between the colon and lungs [20]. Their findings emphasized how inflammation activates microvascular endothelial dysfunction, platelet aggregation, leukocyte–endothelium interactions, and angiogenesis, further illuminating the complex interplay between these organs in the inflammatory process [20].

Epithelia with goblet cells, submucosal glands, and lymphoid tissue protect the host mucosa in the respiratory and gastrointestinal systems, which share the primitive foregut embryology [21]. These common anatomical features suggest that IBD-related epithelial and mucosal immune abnormalities may damage the respiratory tract [22,23]. Epithelial inflammation due to common antigens from inhalation/ingestion (smoke, stress, infections, medicines, nutrition) may cause gastrointestinal and respiratory changes [23].

**Figure 4:**
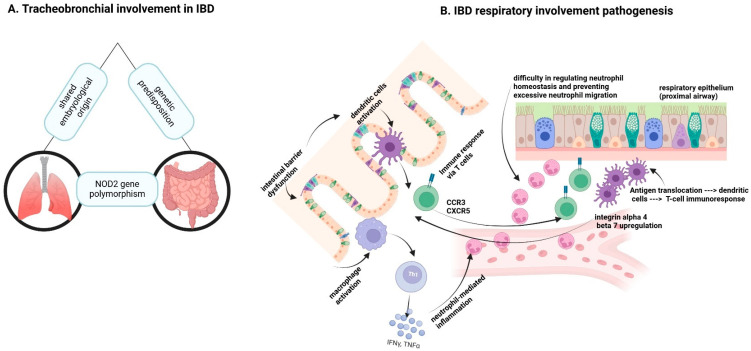
Pathogenesis of respiratory involvement in IBD: from genetic links to immune dysregulation. Created with BioRender.com [17] (accessed on 11 June 2025).

Figure 4 illustrates the mechanisms underlying respiratory involvement in IBD. Panel A highlights tracheobronchial involvement, emphasizing a shared embryological origin and genetic predisposition, such as NOD2 gene polymorphisms, linking lung and intestinal manifestations. Panel B delves into the pathogenesis of IBD-related respiratory involvement. It shows how intestinal barrier dysfunction leads to the activation of dendritic cells, triggering an immune response via T cells, with chemokine receptors CCR3 and CXCR5 playing key roles. This cascade includes macrophage activation, releasing inflammatory mediators like IFN-γ and TNF-α, which further amplify Th1-mediated inflammation. Figure 4 also describes the upregulation of integrin alpha 4 beta 7, facilitating T-cell migration to the respiratory epithelium. This process disrupts neutrophil homeostasis, contributing to excessive neutrophil-mediated inflammation in the proximal airway, illustrating the complex immune interactions between the gut and lungs in IBD.

### 1.4. Rationale for the Review

This review aims to synthesize current evidence on the prevalence, clinical presentation, diagnostic approaches, and outcomes of interstitial lung diseases in IBD patients. Key areas of focus include the clinical manifestations of ILDs, the diagnostic challenges they pose, and their broader implications for managing IBD.

## 2. Materials and Methods

### 2.1. Study Design and Strategy

This study is a systematic review of the literature aimed at exploring the patterns of interstitial lung disease in patients with inflammatory bowel disease, focusing on clinical presentation, diagnostic criteria, and management approaches. This systematic review was conducted in accordance with the Preferred Reporting Items for Systematic Reviews and Meta-Analyses (PRISMA) guidelines. All relevant items from the PRISMA checklist were addressed to ensure transparency, reproducibility, and methodological rigor. A comprehensive literature search was conducted using PubMed database. The literature search was restricted to this database because it provides comprehensive biomedical coverage. While the use of additional databases (e.g., Embase, Scopus) could have broadened the scope, PubMed was selected due to its indexing of core clinical and translational journals.

The search included studies published up to the 1st of May 2025. The following keywords and MeSH terms were used: “inflammatory bowel disease,” “Crohn’s disease,” “ulcerative colitis,” “interstitial lung disease,” “pulmonary manifestations,” “nonspecific interstitial pneumonia,” “cryptogenic organizing pneumonia,” and “pulmonary fibrosis.” To ensure transparency and reproducibility, we provide the complete search strategies used in each database: “Inflammatory Bowel Diseases” [Mesh] OR “Crohn Disease” [Mesh] OR “Ulcerative Colitis” [Mesh] OR “inflammatory bowel disease” [Title/Abstract] OR “Crohn’s disease” [Title/Abstract] OR “ulcerative colitis” [Title/Abstract]) AND “Lung Diseases, Interstitial” [Mesh] OR “Pulmonary Fibrosis” [Mesh] OR “Bronchiolitis Obliterans Organizing Pneumonia” [Mesh] OR “interstitial lung disease” [Title/Abstract] OR “pulmonary manifestations” [Title/Abstract] OR “cryptogenic organizing pneumonia” [Title/Abstract] OR “pulmonary fibrosis” [Title/Abstract] OR “bronchiectasis” [Title/Abstract]).

### 2.2. Inclusion and Exclusion Criteria

Studies were included if they met the following criteria:

- Reported pulmonary manifestations, specifically ILD, in IBD patients.
- Were original research articles, including cohort studies, case-control studies, case series, and case reports.
- Were published in English.

Exclusion criteria included the following:

- Studies focusing solely on pediatric populations.
- Non-human studies.
- Reviews, meta-analyses, and editorials without original patient data.
- Articles lacking sufficient detail on pulmonary involvement in IBD.

### 2.3. Study Selection, Data Extraction, and Synthesis

Titles and abstracts were screened independently by two reviewers. Full-text articles of potentially relevant studies were assessed for eligibility. Discrepancies were resolved through discussion or by consulting a third reviewer. Data were extracted using a standardized form, capturing information on study design, population characteristics, types of ILD, diagnostic methods (e.g., pulmonary function tests, imaging, histopathology), treatments, and outcomes. Findings were synthesized qualitatively, and where possible, quantitative data were summarized.

### 2.4. Quality Assessment and Statistical Analysis

The quality of included studies was assessed using the Newcastle–Ottawa Scale (NOS) for observational studies [24]. Case reports and case series were evaluated using an adapted version of the Joanna Briggs Institute Critical Appraisal Tool [25]. Descriptive statistics to summarize study characteristics were performed using SPSS version 29.0 [26]. Data on ILD prevalence, clinical outcomes, and treatments were analyzed where applicable. Heterogeneity across studies precluded meta-analysis.

**Figure 5:**
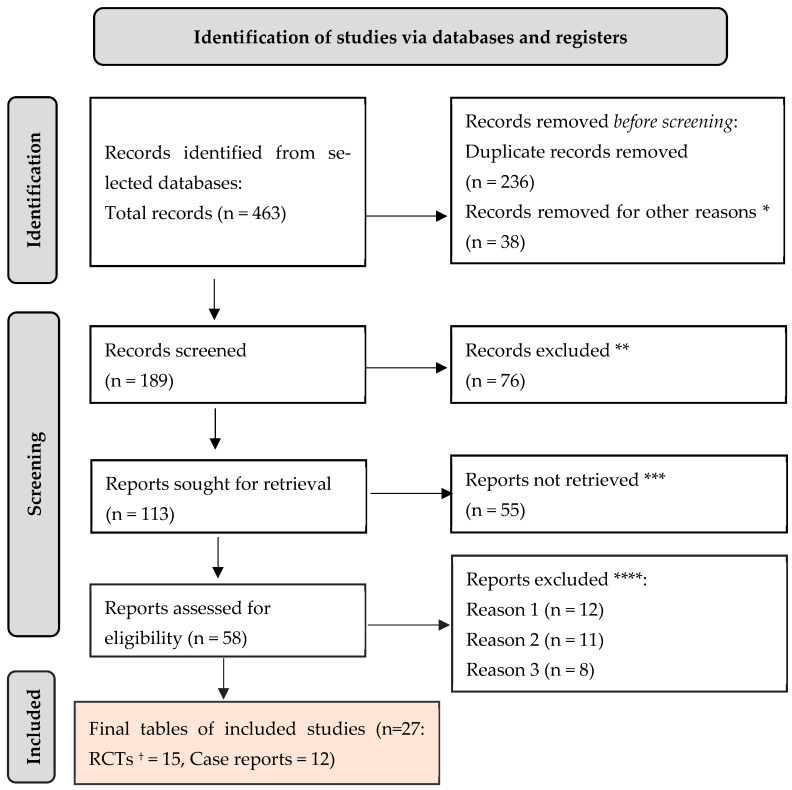
PRISMA framework. * 14 studies investigated the wrong population (e.g., pediatric cohorts or non-IBD populations); 9 studies focused on the wrong intervention or exposure (e.g., treatment studies unrelated to pulmonary manifestations); 11 studies reported outcomes not relevant to pulmonary involvement (e.g., gastrointestinal-only outcomes without respiratory data); 4 studies provided insufficient data for extraction or analysis. ** studies do not help us to provide an answer to the current research. *** unable to find the full text of the study. **** Reason 1—study on animals/Reason 2—wrong setting/Reason 3—research question not relevant. † Randomised controlled trials.

As it can be seen in Figure 5, a total of 463 citations were retrieved after scanning the aforementioned databases. After eliminating duplicate entries (236) and excluding 38 items that did not satisfy the search parameters, the list was reduced to 189 remaining articles. A total of 76 studies were excluded from consideration as they did not meet the requirements of our research, based on their abstracts. Additionally, 55 papers were further eliminated because they did not address the specific question of this study. Furthermore, 12 studies were excluded due to the unavailability of the full text. Another 11 studies were omitted because they focused on the wrong age group. Lastly, 8 articles were disregarded as they were written in a language other than English. Thus, we based our final analysis on a total of 27 search results that met the criteria for our investigation.

## 3. Results

Based on data from the first 15 RCTs, pulmonary involvement in IBD presents significant risks. Jussila et al. [27] reported on 21,964 patients, finding a SMR of 2.01 for respiratory diseases in CD and 1.24 in UC, with 2244 respiratory-related deaths. Mahadeva et al. [28], in a smaller study of 17 patients, highlighted that 76% had bronchiectasis, showcasing a high prevalence of lung complications. Similarly, Jess et al. [29] identified an increased risk of COPD in CD patients, noting 56 and 62 deaths in CD and UC, respectively, emphasizing the importance of smoking cessation. Other studies included in Table 1 report pulmonary conditions often underdiagnosed in IBD patients [30,31,32] Kelly et al. [33] demonstrated that 50% of 10 post-surgical IBD patients experienced bronchiectasis, while Yılmaz et al. [34] found 64.1% of their 39-patient cohort had HRCT abnormalities, predominantly bronchiectasis and emphysema.

Further insights from Mendoza et al. [35] identified pulmonary embolism as a critical complication, more prevalent in UC, reinforcing the need for comprehensive systemic monitoring. Bernstein et al. [32] reported asthma and bronchitis as common comorbidities in an IBD cohort of 8072 patients, emphasizing the often-underdiagnosed nature of pulmonary conditions in IBD. Across these studies, smoking emerged as a key factor, with rates up to 19% (Black et al. [39]), exacerbating conditions such as bronchitis and COPD. These findings collectively accentuate the critical need for proactive respiratory evaluation and management in IBD, particularly given the chronic nature and systemic implications of the disease. 

Table 2 below shows the pulmonary manifestations from the randomised controlled trials included in this review.

Pulmonary manifestations are significant extraintestinal complications in both CD and UC. Bronchiectasis is highly prevalent, affecting 76% of CD patients in Mahadeva et al. [28], with HRCT abnormalities noted in 64.1% of CD cases presented by Yılmaz et al. [34] and 22% via HRCT in Desai et al. [40]. In UC, bronchiectasis was detected in 13% of patients from Mahadeva et al.’s study [28] and 28.5% of cases in Desai et al. [40]. COPD risk is increased in CD, particularly among smokers [29,39]. Pulmonary embolism is more common in CD, contributing to elevated mortality rates, with a standardized mortality ratio of 2.01 in CD versus 1.24 in UC [27,35]. Asthma and bronchitis were frequently reported, especially in Bernstein et al.’s IBD cohorts [32,41].

**Figure 6:**
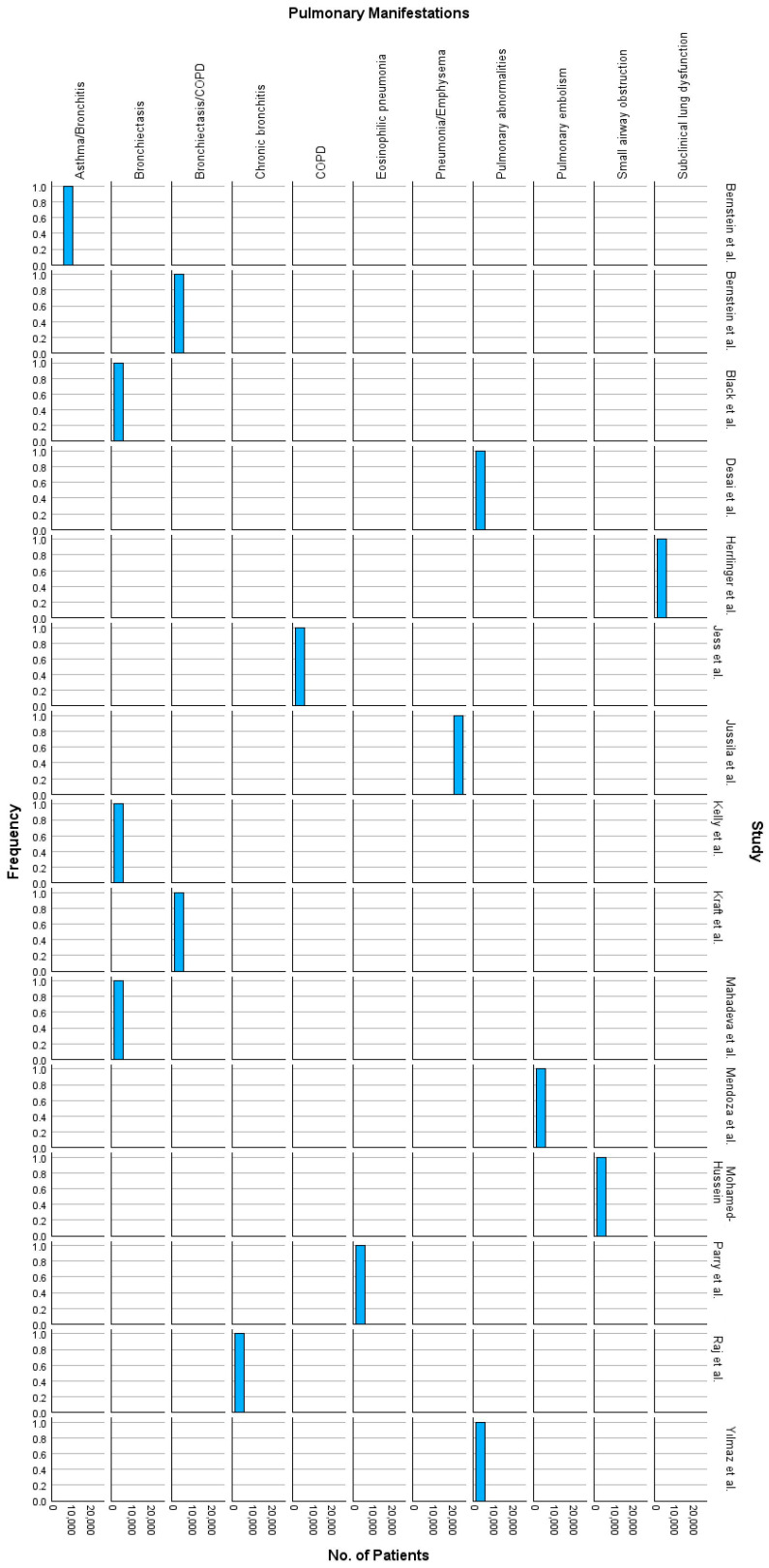
Frequencies of pulmonary manifestations in the 15 RCTs [27,28,29,30,31,32,33,34,35,36,37,38,39,40,41].

Figure 6 shows the distribution of various pulmonary manifestations across the 15 RCTs included in Table 1. The chart highlights the relative frequency of pulmonary conditions among study participants. Notably, bronchiectasis appears as one of the most frequently reported complications, particularly in studies with larger sample sizes, such as Jussila et al. [27] and Bernstein et al. [32]. Similarly, pulmonary embolism and small airway obstruction show a lesser but consistent presence. This visualization features the heterogeneity of pulmonary complications in IBD, with varying prevalence depending on the study population and clinical focus.

Table 3 includes the pulmonary manifestations from the case reports included in this analysis.

The 12 case reports highlight diverse pulmonary manifestations in CD and UC. Pulmonary nodules were common in CD, with necrobiotic nodules reported in multiple cases (Freeman et al. [44]; Golpe et al. [46]), while UC presented pulmonary nodules as an early extraintestinal manifestation in Chew et al. [43]. Granulomatous inflammation was prominent in CD, appearing as granulomatous bronchiolitis or necrobiotic granulomas in over 30% of cases [44,47,50], compared to non-necrotizing granulomas in UC [48]. Organizing pneumonia, a hallmark of pulmonary involvement, was documented in both diseases, with CD showing a granulomatous variant [53] and UC associated with active flares [48]. Eosinophilic pneumonitis was linked to drug therapy in UC (Peters et al. [42]) and interstitial pneumonitis in CD [52]. Pleural involvement, including pachypleuritis and pleural effusions, was noted in both conditions, predominantly in Faller et al. [51] for CD and Peters et al. [42] for UC.

Table 4 contains the frequency of pulmonary manifestations included in the case reports.

The frequency table presented above highlights the distribution of pulmonary manifestations between CD and UC. Granulomatous inflammation (Code 2) is the most frequent manifestation in CD, appearing in three cases, while pulmonary nodules (Code 1) are observed in two cases. Other complications like organizing pneumonia (Code 3), eosinophilic pneumonitis (Code 4), pleural effusion or thickening (Code 5), bronchiectasis (Code 6), and ILD, Code 7) occur less frequently, with bronchiectasis appearing solely in CD. Conversely, UC shows a more even distribution across manifestations, with pulmonary embolism (Code 8) exclusively reported in UC.

**Figure 7:**
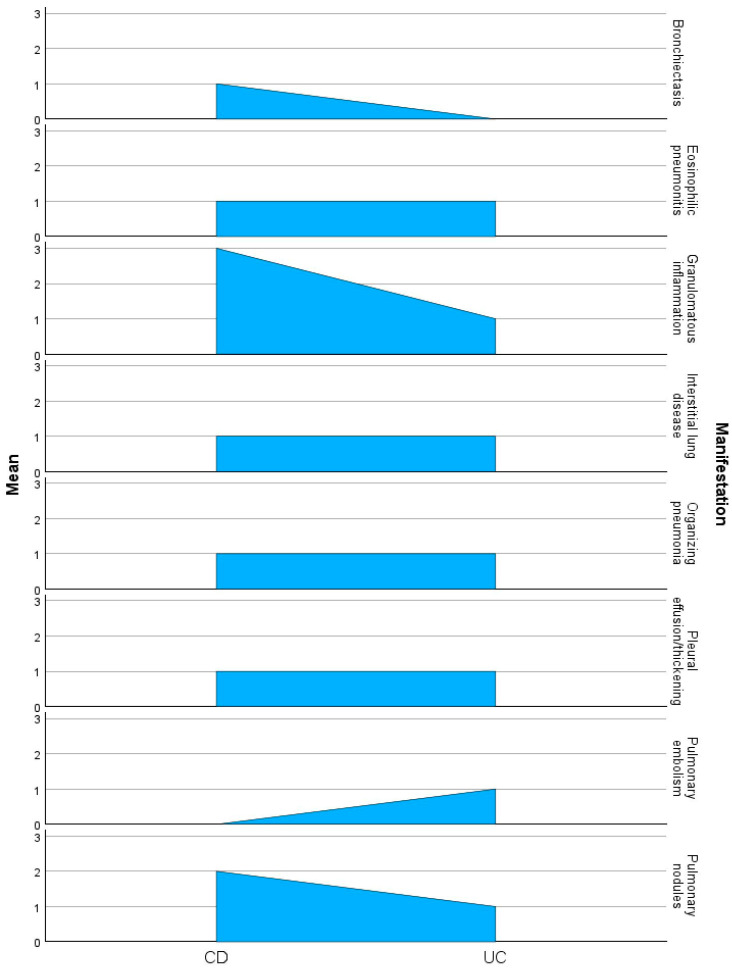
Comparative frequency of pulmonary manifestations in CD and UC, based on the 12 case reports.

Figure 7 illustrates the mean frequency of various pulmonary manifestations observed in CD and UC reported in the 12 case studies included in this analysis. Granulomatous inflammation is the most frequent manifestation in CD, with a frequency of three cases, compared to one case in UC. Pulmonary nodules were reported in two CD cases and one UC case, highlighting their prevalence as a common manifestation. Interestingly, pulmonary embolism was exclusively observed in UC, with a frequency of one case, while bronchiectasis, identified in one case, was limited to CD. Pleural effusion and thickening, along with eosinophilic pneumonitis and organizing pneumonia, were equally distributed, each occurring in one case for both CD and UC.

## 4. Discussion

The present study elucidates the spectrum of pulmonary manifestations in IBD, highlighting their prevalence and clinical implications. Our findings indicate that granulomatous inflammation is the most frequent pulmonary manifestation in Crohn’s disease, observed in three cases, whereas pulmonary nodules are reported in two cases. In contrast, Ulcerative colitis presents a more diverse array of pulmonary complications, including pulmonary embolism, which is exclusively noted in UC cases.

These observations align with the existing literature [54,55,56]. For instance, Mahadeva et al. [28] reported a higher prevalence of bronchiectasis in UC patients compared to CD, suggesting a differential pattern of pulmonary involvement between the two conditions. Similarly, Black et al. [39] documented a variety of pulmonary manifestations in IBD, with bronchiectasis being the most common, particularly in UC patients.

Beyond the epidemiological and clinical findings, several studies suggest other potential mechanistic pathways linking IBD and pulmonary involvement. For example, polymorphisms in the NOD2 gene, widely implicated in CD, have also been associated with altered pulmonary immune responses. Similarly, the overexpression of integrin α4β7 and chemokine receptors such as CCR3 and CXCR5 facilitates T-cell migration from the intestinal to the respiratory epithelium, highlighting shared immunological circuits between the gut and lungs.

The exclusive occurrence of pulmonary embolism in UC patients within our study is noteworthy. Mendoza et al. [35] highlighted an increased risk of thromboembolic events in UC, attributing it to the hypercoagulable state associated with the disease. This is similar to other findings in the literature that emphasize on the necessity for vigilant monitoring and prophylactic measures in UC patients to mitigate such risks [57,58].

Our study also identifies organizing pneumonia, eosinophilic pneumonitis, and pleural effusion/thickening as pulmonary manifestations present in both CD and UC, each occurring in one case [42,48,51,52,53]. These findings are consistent with those of Yilmaz et al. [34], who reported various pulmonary abnormalities in IBD patients, including interstitial lung disease and pleural involvement.

The differential distribution of pulmonary manifestations between CD and UC observed in our study highlights the importance of tailored clinical approaches. Healthcare providers should maintain a high index of suspicion for pulmonary complications in IBD patients, particularly given their potential impact on morbidity and mortality [59].

## 5. Strengths and Limitations

This study offers significant strengths, including a comprehensive analysis of pulmonary manifestations in IBD. By utilizing a diverse sample set and incorporating case reports alongside randomized controlled trials, it provides a broad overview of pulmonary complications across different IBD subtypes. Another strength is the detailed comparison with the existing literature, which contextualizes our findings within a wider clinical framework.

However, several limitations must be acknowledged. The findings of several case studies may be limited by their small sample size. Some included studies are retrospective, which may involve selection bias and missing data. Moreover, reliance on a single database (PubMed) may have excluded relevant studies indexed elsewhere.

Another limitation of our review is the heterogeneity in the way primary studies reported their data. For example, some studies presented patient age as a mean or median, while others provided only ranges. Similarly, sex distribution, smoking status, and outcomes were reported variably as absolute numbers or percentages. We standardized the data presentation as much as possible (e.g., converting counts to percentages and presenting age consistently as mean or median when reported). However, in cases where the original studies did not provide sufficient detail for conversion, we retained the data in the format available.

Finally, our study only identifies crucial pulmonary symptoms, not pathophysiological mechanisms. Future prospective studies with larger cohorts are needed to confirm these findings and study IBD-pulmonary problems causal links.

## 6. Conclusions

This study highlights the diverse spectrum of pulmonary manifestations in IBD, emphasizing their clinical significance in both CD and UC. Granulomatous inflammation emerged as the most frequent pulmonary complication in CD, while pulmonary nodules were common in both conditions. Notably, pulmonary embolism was exclusively observed in UC, underscoring its unique thromboembolic risk. These findings align with the existing literature, supporting the role of chronic inflammation and hypercoagulability in pulmonary complications.

Additionally, rare but significant manifestations, such as organizing pneumonia, eosinophilic pneumonitis, and pleural effusion, were identified in both CD and UC, highlighting the importance of recognizing less common presentations. This study features the need for routine pulmonary evaluation in IBD patients to ensure early detection and management of respiratory complications. Future research should focus on larger, prospective studies to confirm these findings and explore the underlying mechanisms linking IBD to pulmonary involvement.

## Figures and Tables

**Table 1:** Summary of key studies and case reports.

Study	Patients (*n*)	Age (Years)	Male (%)	Other Population Characteristics	Smoking (*n*)	Pulmonary Involvement	Key Findings	Deaths (*n*)	Conclusion	Years from Onset
**Randomised controlled trials**										
Jussila et al. (2014) [27]	21,964	40	48%	Nationwide Finnish IBD cohort; 16,649 UC, 5315 CD	Not reported	pulmonary embolism, pneumonia, bronchitis, emphysema	SMR * for respiratory diseases: 2.01 in CD, 1.24 in UC; pneumonia & COPD * common	2244	Chronic inflammation contributes to elevated mortality in IBD	1–25
Mahadeva et al. (1999) [28]	17	38–83	58.8%	14 UC, 3 CD patients	7 ex-smokers, 3 current smokers, 7 never smoked	13 patients with bronchiectasis (76%), 9 patients with air trapping, 5 with “tree in bud” pattern, peripheral reticular changes	Pulmonary abnormalities responsive to steroids; concurrent UC and lung exacerbations in some cases	Not reported	Pulmonary involvement in IBD is varied and steroid-responsive in many cases	1–25
Jess et al. (2006) [29]	692	33.3	53.03%	314 CD, 378 UC; Olmsted County population-based cohort	Smoking prevalent in CD	Increased risk of COPD in CD	CD mortality from GI disease and COPD; UC mortality lower than general population	56 (CD), 62 (UC)	Long-term outcomes stable; smoking cessation crucial in CD to reduce COPD risks	14
Parry et al. (2002) [30]	50	48.3	66%	72% UC, 6% CD, various arthritis cases	Data not provided	Eosinophilic pneumonia, interstitial fibrosis	Pulmonary toxicity from sulphasalazine, symptoms improved post drug withdrawal in most cases	5	Pulmonary toxicity rare, resolves post drug withdrawal, corticosteroids debatable in treatment	0.5–10
Mohamed-Hussein et al. (1996) [31]	26	39.5	65%	20 active UC, 6 inactive UC patients	3 smokers (11.5%)	Small airway obstruction, restrictive and obstructive dysfunction	Pulmonary dysfunction common, more pronounced in active UC, no smoking impact on PFTs	0	Early recognition of lung issues is key; PFTs improve with treatment	1–3
Bernstein et al. (2005) [32]	8072	42.5	49%	Manitoba IBD cohort: 3879 UC, 4193 CD	Smoking more prevalent in CD patients	Asthma and bronchitis common as comorbidities	Increased prevalence of asthma and bronchitis among IBD patients compared to controls	Not reported	Pulmonary conditions often underdiagnosed in IBD patients	Not specified
Kelly et al. (2006) [33]	10	56		5 UC, 5 CD; post-surgical patients	3 smokers, 4 ex-smokers	Bronchiectasis, small airways disease	Surgery for IBD associated with respiratory symptoms, particularly bronchiectasis	0	Pulmonary symptoms often develop post-IBD surgery	9–35
Yılmaz et al. (2010) [34]	39	44.3	59%	30 UC, 9 CD, Turkey cohort	4 smokers, 5 ex-smokers	Peribronchial thickening, bronchiectasis, emphysema	64.1% had HRCT abnormalities; pulmonary symptoms correlated with active bowel disease	0	Early detection and management of respiratory symptoms in IBD is crucial	0–9
Mendoza et al. (2005) [35]	566	34.5	53.5%	Spanish cohort: 295 CD, 271 UC	12% smokers overall	Rare but documented in both CD and UC	Pulmonary embolism more common in UC; extraintestinal manifestations frequent	Not reported	Comprehensive monitoring needed for systemic complications in IBD	2–32
Kraft et al. (1976) [36]	6	45–59	50%	5 UC, 1 CD, severe cases	4 non-smokers, 2 ex-smokers	Chronic bronchitis, bronchiectasis, COPD	Pulmonary symptoms persisted post-colectomy in 2 cases; bronchiectasis linked to IBD	2	Pulmonary diseases are a systemic complication in IBD	3–13
Raj et al. (2008) [37]	37	61	45%	Airways disease patients with IBD (22 UC, 13 CD)	5 current smokers, 16 ex-smokers	Chronic bronchitis, bronchiectasis	IBD prevalence higher in airways disease; non-asthmatic airway disease common in IBD	Not reported	Airways disease frequently coexists with IBD, especially UC	15–16
Herrlinger et al. (2002) [38]	66	36	47%	35 CD, 31 UC; Germany cohort	15% smokers overall	Reduced FEV1 *, IVC *, DLCO * in 42%	Pulmonary dysfunction linked to disease activity, persists in remission	Not reported	Subclinical lung dysfunction is a frequent, persistent extraintestinal IBD manifestation	1–29
Black et al. (2007) [39]	155	45	40%	Cases from 55 English-language reports; UC predominates	19% current smokers	Bronchiectasis most common, BOOP, airway hyperresponsiveness	Pulmonary involvement spans airways to pleura; varied	Not reported	Screening studies reveal high prevalence of respiratory issues in IBD	2–20
Desai et al. (2011) [40]	95	41.9	50.5%	83 UC, 12 CD; Mumbai cohort	Smoking prevalence low	Bronchiectasis, nodules, emphysema	28.5% had PFT abnormalities; HRCT detected pulmonary changes in 22%	0	Routine HRCT for IBD patients may detect latent pulmonary disease	1–30
Bernstein et al. (2001) [41]	4454	40	48%	Manitoba IBD Database (population-based cohort)	Not reported	COPD, bronchiectasis	Pulmonary conditions more common in IBD patients than in general population	Not reported	Early recognition of extraintestinal manifestations critical in IBD management	1–20
**Case reports**										
Peters et al. (1997) [42]	1	65	N/A	Patient with rheumatoid arthritis, treated with sulphasalazine	Non-smoker	Eosinophilic pneumonitis	Symptoms (dyspnoea, fever) resolved after sulphasalazine withdrawal	0	Sulphasalazine-induced pulmonary toxicity is rare and reversible upon drug cessation	<1
Chew et al. (2016) [43]	1	44	N/A	Female with Crohn’s Disease	Non-smoker	Pulmonary nodules, necrotizing granulomas	Pulmonary symptoms led to Crohn’s diagnosis; lung disease as EIM * of IBD	0	Pulmonary EIM can precede gastrointestinal symptoms in Crohn’s disease	<1
Freeman et al. (2004) [44]	1	37	N/A	Extensive Crohn’s (stomach, small, large intestine)	Non-smoker	Granulomatous bronchiolitis, necrobiotic nodules	Pulmonary involvement resolved with steroids; necrotizing nodules linked to Crohn’s	0	Pulmonary manifestations are rare but manageable with immunosuppression	~10
Garg et al. (2020) [45]	1	41	N/A	Female with Crohn’s, post-colectomy	Non-smoker	Ground-glass opacities, pulmonary nodules	Pulmonary symptoms resolved with steroids; nodules linked to Crohn’s as EIM	0	Pulmonary involvement in Crohn’s should be considered in unexplained lung diseases	9
Golpe et al. (2003) [46]	1	68	N/A	Female with long-standing Crohn’s Disease	Non-smoker	Multiple pulmonary nodules, Nongranulomatous lymphoid infiltration	Pulmonary symptoms resolved with steroids; nodules linked to CD	0	Pulmonary nodules in Crohn’s patients may mimic malignancy; respond to steroids	14
Ghosh et al. (2023) [47]	1	73	N/A	Female with GERD * and liver haemangiomas; first pulmonary sign of Crohn’s	Non-smoker	Pulmonary nodules, granulomatous inflammation	Pulmonary nodules resolved with infliximab; EIMs preceded GI symptoms	0	Pulmonary involvement can precede GI symptoms in CD; thorough work-up is crucial	<1
Haenen et al. (2023) [48]	1	30	N/A	UC, recent flare, history of immunosuppressive therapy	Non-smoker	Cavitary necrobiotic pulmonary nodules	Nodules resolved with steroids and ustekinumab; lung symptoms mirrored UC flare	0	Pulmonary manifestations in UC can mimic malignancy, need biopsy for diagnosis	~0.5
Shtaya et al. (2021) [49]	1	31	N/A	Crohn’s disease under vedolizumab therapy	Non-smoker	Pulmonary granulomas, pleural effusion	Lung findings resolved with infliximab; granulomas linked to Crohn’s as EIM	0	Vedolizumab may unmask pulmonary EIM; infliximab effectively treats pulmonary symptoms	13
Warwick et al. (2009) [50]	1	22	N/A	Female with Crohn’s disease and anterior uveitis	Non-smoker	Necrobiotic pulmonary nodules	Nodules responded to systemic steroids; rare pulmonary manifestation of Crohn’s	0	Necrobiotic nodules are rare EIMs of Crohn’s and respond well to steroids	Newly diagnosed
Faller et al. (2000) [51]	1	38	N/A	Female with Crohn’s disease and eosinophilia	Non-smoker	Migratory infiltrates, pachypleuritis, necrotizing nodules	Pulmonary symptoms resolved with corticosteroids; associated with Crohn’s disease or mesalazine use	0	Pulmonary manifestations in Crohn’s can mimic infections; steroids are effective	3
Hotermans et al. (1996) [52]	1	33	N/A	Female with Crohn’s disease on mesalazine	Non-smoker	Nongranulomatous interstitial lung disease	Significant improvement with corticosteroids and cyclophosphamide; worsened without steroids	0	ILD in Crohn’s can mimic IPF *; immunosuppression effective	3
Athayde et al. (2018) [53]	1	34	N/A	Male with severe intestinal Crohn’s Disease, treated with infliximab	Non-smoker	Organizing pneumonia with granulomatous inflammation	Symptoms resolved with corticosteroids and immunomodulation with azathioprine and infliximab	0	Pulmonary manifestations are rare but treatable with steroids and immunosuppressants	2

* SMR: Standardized mortality ratio; COPD: chronic obstructive pulmonary disease; FEV1: forced expiratory volume; IVC: inspiratory vital capacity; DLCO: diffusing capacity of the lungs for Carbon Monoxide; EIM: extraintestinal manifestation; GERD: gastroesophageal reflux disease; IPF: idiopathic pulmonary fibrosis.

**Table 2:** Pulmonary manifestations from the 15 RCTs.

Pulmonary Manifestation	Crohn’s Disease	Ulcerative Colitis
Bronchiectasis	76% of patients [28]	13% of patients [28]
	Post-surgical CD patients [33]	Post-surgical UC patients (Kelly et al. [33])
	64.1% showed HRCT abnormalities [34]	Detected in 28.5% via HRCT [40]
	Detected in 22% via HRCT [40]	Reported cases in IBD cohort [41]
	Pulmonary conditions more common in CD [41]	
COPD	Increased risk of COPD [29]	Less prevalent than CD, but reported [29]
	Exacerbated by smoking [39]	
Interstitial lung disease	Eosinophilic pneumonia, fibrosis [30,31]	ILD less frequent [30]
Pulmonary embolism	More common in CD [27,35]	SMR 1.24 for respiratory diseases [27]
Asthma and Bronchitis	Frequently reported [27,32]	Common comorbidities [27,32]
Pneumonia, emphysema	SMR 2.01 [27]	SMR 1.24 [27]

**Table 3:** Pulmonary manifestations from the 12 case reports.

Pulmonary Manifestation	Crohn’s Disease	Ulcerative Colitis
Pulmonary nodules	Necrobiotic nodules reported [44,46]	Pulmonary nodules preceding GI symptoms [43]
Granulomatous inflammation	Granulomatous bronchiolitis, necrobiotic granulomas (Freeman et al. [44,47,50]	Non-necrotizing granulomas in lung biopsy [48]
Organizing pneumonia	Organizing pneumonia with granulomatous inflammation [53]	Pulmonary organizing pneumonia linked to UC flare [48]
Eosinophilic pneumonitis	Interstitial pneumonitis, eosinophilic infiltrates [52]	Eosinophilic pneumonitis linked to sulfasalazine therapy [42]
Pleural effusion/thickening	Migratory infiltrates, pachypleuritis [51]	Bilateral pleural thickening linked to UC flare [42]
Bronchiectasis	Noted in some CD patients post-colectomy [45]	Not explicitly reported in case reports
Interstitial lung disease	Nongranulomatous ILD reported; improved with immunosuppression [52]	Rare ILD cases linked to UC [43]
Pulmonary embolism	None reported in case studies	Pulmonary embolism rare but recognized (generalized in studies, not specific case)

**Table 4:** Frequency table for pulmonary manifestations included in the 12 case reports.

Pulmonary Manifestation	Code	Frequency in CD	Frequency in UC
Pulmonary nodules	1	2	1
Granulomatous inflammation	2	3	1
Organizing pneumonia	3	1	1
Eosinophilic pneumonitis	4	1	1
Pleural effusion/thickening	5	1	1
Bronchiectasis	6	1	0
Interstitial lung disease	7	1	1
Pulmonary embolism	8	0	1

## Data Availability

No new data were generated.
